# Classification of tumours

**DOI:** 10.1186/1756-9966-27-70

**Published:** 2008-11-14

**Authors:** Helge L Waldum, Arne K Sandvik, Eiliv Brenna, Reidar Fossmark, Gunnar Qvigstad, Jun Soga

**Affiliations:** 1 Norwegian University of Science and Technology, Department of Cancer Research and Molecular Medicine, Trondheim University Hospital, NO-7006 Trondheim, Norway; 2 Niigata University, Niigata, Japan

## Abstract

Tumours are classified according to the most differentiated cells with the exception of carcinomas where a few tumour cells show neuroendocrine differentiation. In this case these cells are regarded as redifferentiated tumour cells, and the tumour is not classified as neuroendocrine. However, it is now clear that normal neuroendocrine cells can divide, and that continuous stimulation of such cells results in tumour formation, which during time becomes increasingly malignant. To understand tumourigenesis, it is of utmost importance to recognize the cell of origin of the tumour since knowledge of the growth regulation of that cell may give information about development and thus possible prevention and prophylaxis of the tumour. It may also have implications for the treatment. The successful treatment of gastrointestinal stromal tumours by a tyrosine kinase inhibitor is an example of the importance of a correct cellular classification of a tumour. In the future tumours should not just be classified as for instance adenocarcinomas of an organ, but more precisely as a carcinoma originating from a certain cell type of that organ.

## 

During tumourigenesis cellular phenotype changes as mutations occur and accumulate. Most mutations result in functional loss. However, when mutations affect an inhibitory mechanism, the net result may be a gain of function. Typically in malignant cells such a loss results in increased proliferation and/or gain in the ability to invade surrounding structures or to spread from the tissue of origin (metastasize). In order to grow beyond a critical cell mass new blood vessels have to develop in the tumour (angiogenesis). Morphologically, tumour cells change their appearance at the cytological level (cellular atypia) and their growth pattern (dysplasia). Such gradual changes are typically seen in tumours of the colon where adenomas with different degrees of dysplasia or overt carcinomas are seen [1], occasionally in the same patient. Similarly, a gradual transition from normal to dysplastic to malignant growth can be seen in the enterochromaffin like (ECL) cells in the oxyntic mucosa [2] particularly after long-term hypergastrinemia in man [3] as well as animals [4]. It is well-known that the cells of highly malignant tumours may have changed so much that the cell of origin may be difficult to recognize in so-called dedifferentiated or anaplastic tumours. Often, however, parts of the tumour are more differentiated, and it is generally accepted that a tumour is classified according to its most differentiated part. There is, however, one exception to this classification, namely when a few neuroendocrine tumour cells are found among dedifferentiated and anaplastic tumour cells [5]. In such cases the tumours are classified according to the presumed origin of the majority of the cells, and the neuroendocrine differentiated tumour cells are neglected. These neuroendocrine tumour cells are considered to be the result of a neuroendocrine redifferentiation of an exocrine derived tumour cell [6,7].

## Neuroendocrine tumours

Neuroendocrine tumours are generally difficult to classify into benign or malignant tumours [8]. Tumours with a highly differentiated and almost normal phenotype may prove to be malignant based upon the detection of local invasion or metastasis, which may be found years after removal of the initial tumour [9]. Thus, neuroendocrine tumours may morphologically look rather benign and grow slowly, but nevertheless they have the ability to metastasize. This property may be explained by an effect of their normal mediators on the surrounding tissues. Thus, these mediators like for instance histamine from the ECL cell, may dilate the vascular bed and at the same time increase the permeability of the capillaries making it easier for the tumour cells to gain access to the blood [10]. Moreover, histamine and other substances from neuroendocrine cells have a stimulatory effect on angiogenesis [11]. In other words, many of these neuroendocrine cells normally produce substances making it easier for these cells to grow and disseminate. This may also explain why such tumours may not need so many mutations and accordingly not be so changed phenotypically as other tumours, while still having the ability to metastasize.

In many tumours classified as adenocarcinomas based upon a glandular growth pattern and/or tumour cells positive for PAS or Alcian blue and thus believed to contain mucin, there are tumour cells with neuroendocrine properties [12]. These neuroendocrine cells are believed to be redifferentiated exocrine derived tumour cells [6,7]. Consequently, these cells do not have any implication for the classification of the tumour. However, when increasing the sensitivity of immunohistochemistry by tyramide signal amplification (TSA) [13], the number of chromogranin A (CgA) positive tumour cells are greatly increased [14]. CgA occurs exclusively in the neuroendocrine granules and such immunoreactivity is thus specific for neuroendocrine derived cells [15]. Thus, neuroendocrine tumour cells in anaplastic tumours may represent the most differentiated tumour cells in the dedifferentiated neuroendocrine tumour. If so, these tumours must have their origin in neuroendocrine cells, which have gradually become more malignant. In the oxyntic mucosa of the stomach the ECL cell has a key position in the regulation of acid secretion [16], and this cell is regulated both functionally as well as trophically by gastrin [16,17]. Tumours developing from the ECL cell are classified into three types according to presumed aetiology: groups I and II being related to hypergastrinemia whereas group III tumours apparently occur in normogastrinemic individuals [18]. Although group I ECLomas also obviously may become highly malignant [3], it is clear that the type III ECLomas generally are more malignant than those occurring in hypergastrinemic individuals [18]. This indicates that the ECL cells of the type III ECLomas must have become less gastrin dependent than normal ECL cells [19]. Nevertheless, ECL cell derived tumours induced by chronic hypergastrinemia [20] may also become increasingly malignant [3], and the gastric adenocarcinomas in patients with hypergastrinemia secondary to severe chronic atrophic gastritis may have been derived from the ECL cell and thus be misclassified neuroendocrine carcinomas [21]. The gastric carcinomas in patients with pernicious anaemia could be classified as neuroendocrine tumours after immunohistochemistry for CgA with the help of TSA technique [21]. The TSA technique improves the sensitivity without reducing the specificity since this depends on the specificity of the primary antibody only. Moreover, the signet ring tumour cells in gastric carcinomas, believed to be mucin producing, are positive for neuroendocrine markers [22]. It is worth recalling that PAS staining is not specific for mucin, since glycoproteins in general are stained by PAS [23]. Furthermore, many neuroendocrine cells normally, or in pathological conditions [24], produce glycohormones which may be classified as glycoproteins or glycopeptides. Finally, the secretogranins, being exclusively expressed in neuroendocrine cells, are also glycoproteins [25]. Accordingly, there is a misunderstanding that PAS positivity is specific for exocrine cells. Reclassification of malignant gastric tumours from adenocarcinomas of presumptive of exocrine origin into malignant neuroendocrine carcionomas has previously been done in the tumours occurring spontaneously in mastomys [26], in rodents after life-long hypergastrinemia due to loxtidine dosing [27,28] and in man [29].

Many do not realize that neuroendocrine cells are multipotent [30]. Transformation of neuroendocrine cells in the bronchi into a squamous epithelium has been described in Syrian golden hamsters [31]. Moreover the squamous epithelial cancer cells in the bronchi may show neuroendocrine differentiation [32], suggesting that the two types of bronchial carcinomas most closely related to tobacco smoking, small cell and squamous cell carcinomas [33], both may originate from neuroendocrine cells. Thus, the tumourigenic substance(s) in tobacco smoke, which is neither nicotine [34] nor CO [35], may have its main effect on the bronchial neuroendocrine cells. Hitherto, the prevailing theory has been that neuroendocrine cells and tumours from neuroendocrine cells are derived from stem cells that differentiate into neuroendocrine cell type. However, it is well known that rodent neuroendocrine cells do proliferate [36] and also in man it is obviously difficult to explain how clusters of neuroendocrine cells (micro-carcinoids) far from the proliferation zone of exocrine cells, can occur without self-replication. Moreover, we have been able to describe self-replication of neuroendocrine cells in cancer cells both in the prostate [37] and gastric carcinomas [14]. Very recently, indeed adult beta-cells of the islets of Langerhans were described to be formed by self-duplication rather than differentiation from stem-cell [38].

## Classification of the cell of origin in cancers

To make a correct classification of cellular origin of tumours it is necessary to use immunohistochemistry with antibodies directed against antigens with high specificity, and apply the methods with the highest sensitivity. Is it of importance to make a correct cellular classification of tumours? If the correct cellular classification of tumours does not give any indication of prognosis or treatment, it may be argued that the effort is pointless. This is, however, not true, since a correct cellular classification indicates the pathogenesis of the tumour and thus may be important for the prevention of the tumour based upon knowledge of the growth regulation of the cell of origin. Since the more differentiated type I gastric carcinoids are the most common type and gastrin plays such a dominant role in the trophic regulation of the ECL cell, hypergastrinemia will be expected to increase most tumours of ECL cell origin. Only ECL cell tumours with very early and activating mutations in the gastrin receptor can be assumed to be completely gastrin independent. What the case is in the so-called ECL cell tumours of type III [18] remains to be shown. On the other hand, somatostatin acting via the somatostatin subtype 2 receptor has a negative trophic effect on the ECL cell, which may be used to treat ECL cell derived carcinoids at an early stage [39]. Furthermore, gastrointestinal stromal tumours (GIST) derived from cells of Cajal, possibly being of neural crest origin, may now be treated rather successfully with an inhibitor of a kinase [40], which regulates the proliferation of the Cajal cells. After such treatment became available, these types of tumours have been diagnosed more often. This is an example of a change in classification after an effective treatment of a sub-group of tumours has been recognized. Vice versa, a correct cellular classification of tumours in the future, including knowledge about genetic and cellular mechanisms regulating the growth of the cells, may be expected to tailor drug development and thus give rise to new treatment modalities.

In conclusion, a correct classification of the cell of origin of carcinomas will greatly increase our biological understanding of carcinogenesis, give rise to new possibilities for prevention and early treatment, and possibly to tailor new drugs for the treatment of the tumours.

## Competing interests

The authors declare that they have no competing interests.

## Authors' contributions

The manuscript is based upon the works of two separate groups; JS in Japan working in the area of pathology devoting his life to neuroendocrine pathology, and HLW, Norway starting within physiology, showing the central role of the ECL cell in gastric physiology and transforming this knowledge into pathology. AKS, EB, RF and GQ have taken part in these studies during a long time: AKS in physiology (isolated rat stomach) and molecular medicine, EB in physiology (aminopyrine uptake in isolated parietal cells) and trophic studies, RF in animal tumour models (Japanese cotton rat) and electron microscopy and GQ in gastric physiology in man and pathology in animals and man. All authors have read and approved the final manuscript.
